# Increasing Trends in Opioid Use From 2010 to 2018 in the Region of Valencia, Spain: A Real-World, Population-Based Study

**DOI:** 10.3389/fphar.2020.612556

**Published:** 2020-12-11

**Authors:** Isabel Hurtado, Aníbal García-Sempere, Salvador Peiró, Gabriel Sanfélix-Gimeno

**Affiliations:** ^1^ Fundación Para el Fomento de la Investigación Sanitaria y Biomédica de la Comunidad Valenciana (FISABIO), València, Spain; ^2^ Red de Investigación en Servicios de Salud en Enfermedades Crónicas (REDISSEC), València, Spain

**Keywords:** real-world data, prescription, trends, opioids, morphine milligram equivalents

## Abstract

**Background:** The opioid epidemic has been extensively documented in the United States and Canada, but fewer data are available for Europe.

**Aim:** To describe the trends in opioid use—volume of prescriptions, dosage and number of patients treated—in a Spanish population with more than 4.2 million inhabitants aged 18 years and older.

**Patients and Methods:** Population-based cross-sectional analysis of opioid prescription in adults (≥18 years) from January 1, 2010 to December 31, 2018 in the region of Valencia, Spain. Outcomes were estimated on an annual basis: number of prescriptions, prescription rate per 100 inhabitants, dosage per capita (morphine mg equivalents, MME/c) and volume of patients treated (overall and by drug).

**Results:** Over the study period, 2,107,756 unique patients were prescribed more than 35 million total treatments. The yearly number of treatments doubled, and total MME/c showed almost a threefold increase. Fentanyl MME/c more than tripled, accounting for 34.4% of the total MME/c in 2018. Oxycodone MME/c showed a 10-fold increase, while tapentadol, launched in 2011, showed the highest growth rates. The annual number of patients receiving at least one opioid prescription more than doubled, from 335,379 in 2010 to 722,838 in 2018.

**Conclusions:** Even if proportions still seem far from epidemic, urgent research is warranted on the observed patterns of use, their appropriateness and their association with health and safety outcomes, especially for high-use and high-strength drugs.

## Introduction

Opioid use and misuse are considered a problem of epidemic proportions in the United States (Han et al., 2017; President’s Commission on Combating Drug Addiction and the Opioid Crisis, 2017). In contrast, Spain has traditionally shown one of the lowest rates of opioid consumption in the developed world, far from some countries in Central Europe, the United Kingdom or Canada (García del Pozo et al., 1999; Schubert et al., 2013; Cooper et al., 2017; Fischer et al., 2018). However, in the last 2 decades opioid use has seen unprecedented growth. According to the Spanish Agency for Medicines and Health Products (*Agencia Española de Medicamentos y Productos Sanitarios*, AEMPS), the Spanish National Health System reimbursed 1.3 defined daily doses (DDD) per 1,000 inhabitants/year in 2000, but this figure had increased 15-fold by 2018, reaching 20.0 DDD per 1,000 inhabitants per year (Agencia Española de Medicamentos y Productos Sanitarios, 2008; Agencia Española de Medicamentos y Productos Sanitarios, 2017; Agencia Española de Medicamentos y Productos Sanitarios, 2019). Tramadol, alone or in combination with other analgesics (60%), and fentanyl (15%) accounted for 75% of the DDD consumption, while the use of morphine has remained relatively stable. Recently, the use of newer additions to the therapeutic arsenal, such as tapentadol or oxycodone plus naloxone, seems to be accelerating (Agencia Española de Medicamentos y Productos Sanitarios, 2019).

Although effective pain relief is an essential component of quality health care, these fast-paced trends have raised concerns. In response, the Ministry of Health has published clinical practice guidelines (Ministerio de Sanidad and Servicios Sociales e Igualdad, 2015), the AEMPS has issued significant warnings (Agencia Española de Medicamentos y Productos Sanitarios, 2018), and some regional health departments have developed review utilization interventions, usually targeting the use of immediate-release formulations. Yet, in general there is a lack of real-world information about the patterns of use and misuse of opioids in our setting, limiting the design and adoption of health policies and clinical strategies that ensure adequate control of chronic pain while minimizing the adverse effects of opioids. The aim of this study was to describe the trends in opioid use from 2010 to 2018—in terms of volume of prescriptions, dosage and patients exposed—in a Spanish region with an adult population of more than four million inhabitants.

## Methods

### Design

**FIGURE 1:**
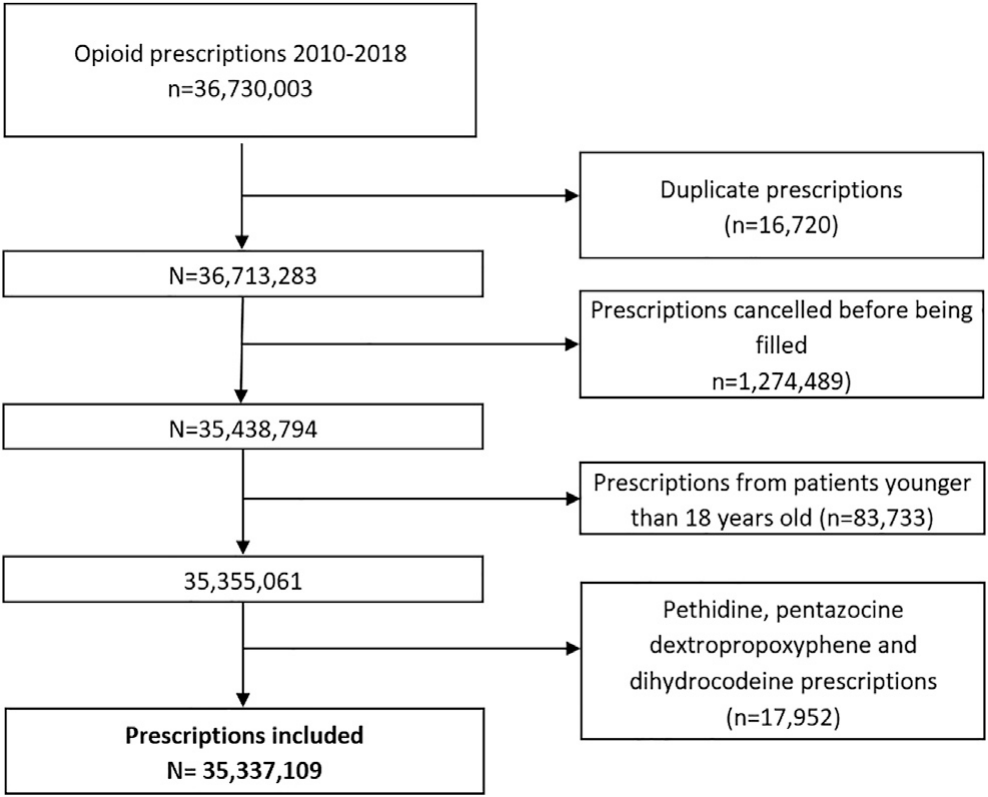
Flowchart.

Population-based, cross-sectional study of all prescriptions for an opioid drug in patients aged 18 and over from January 1, 2010 to December 31, 2018. Opioids included were codeine, tramadol, buprenorphine, fentanyl, hydromorphone, morphine, oxycodone and tapentadol. Use of pethidine, pentazocine, dextropropoxyphene monotherapy (marketing suspension in 2010), and dihydrocodeine was negligible (less than 0.05%), so these drugs were excluded from the analysis (Figure 1). Methadone is not available as a prescription drug in Spain (its use is restricted to detoxification clinics) and was also excluded from the analysis.

### Setting and Population

The study took place in the region of Valencia (Spain) and, specifically, in the population covered by the public Valencia Health System (VHS), which comprises about 97% of the region’s inhabitants. We included all adult patients (≥18 years) who received at least one opioid prescription from January 1, 2010 to December 31, 2018, regardless of whether they were prevalent or new users. Prescription of strong opioids in Spain is regulated by a specific policy mandating prior authorization for the prescription of narcotics. Both specialists and primary care physicians can prescribe them, but once a prescription is issued, indication and adequacy are reviewed before authorization. Dispensation is also subject to a tight, formal control and registry. People without VHS healthcare coverage (mainly certain Spanish government employees whose prescriptions are reimbursed by mutual societies for civil servants, and are thus not included in the pharmacy databases of the VHS), and patients not registered in the municipal census (non-residents or temporary residents), were excluded because of limitations on follow-up. Due to the high population coverage of the VHS, the population denominators were obtained from the census. The size of the reference population remained quite stable, oscillating from 4.1 to 4.2 million adults over the study period (see Supplementary Material S1).

### Data Sources

Data were obtained from the VHS Integrated Databases (VID). The VID is the result of the linkage, by means of a single personal identification number, of a set of publicly owned, population-based healthcare, clinical and administrative electronic databases in Valencia, which has provided comprehensive information for the region’s five million inhabitants since 2008. The VID includes sociodemographic and administrative data (sex, age, nationality) as well as healthcare information such as diagnoses, procedures, laboratory data, pharmaceutical prescriptions and dispensing (including brand and generic name, formulation, strength, and dosing schedule/regimen), hospitalizations, mortality, healthcare utilization and public health data. The VID also includes a set of specific associated databases with population-wide information on significant care areas such as cancer, rare disease, vaccines and imaging data (García-Sempere et al., 2020).

### Outcome Measures

Primary outcomes measures were estimated on an annual basis: number of prescriptions; rate of prescriptions per 100 inhabitants; prescribed dose per capita, measured as morphine milligram equivalents (MME/c; see conversion factors in Supplementary Material S2); and number of patients receiving at least one opioid prescription. All outcomes are presented as totals and by opioid.

### Analysis

We undertook a descriptive analysis of the yearly volume of prescriptions, the rate of prescriptions per 100 inhabitants per year, the dosage of prescriptions in terms of MME per capita and year, and the annual number of patients treated with at least one opioid prescription from 2010 to 2018.

## Results

**FIGURE 2:**
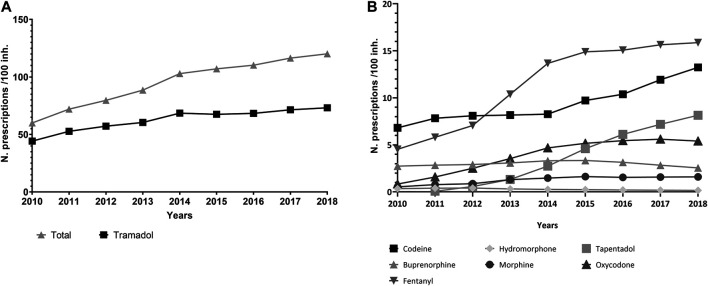
**(A,B)** Trends in the rate of prescriptions per 100 inhabitants, period 2010–2018, region of Valencia, Spain.

More than 35 million treatments were prescribed in total (Figure 1), and the number of treatments per year nearly doubled over the study period, from 2,524,459 prescriptions in 2010 to 4,894,125 in 2018 (Table 1). Tramadol (alone or in combination with other non-opioid analgesics) accounted for 65.8% of the total volume of prescription in the period, and fentanyl, 12.0%. Tramadol use increased from 44.3 prescriptions/100 inhabitants in 2010 to 71.5 prescriptions/100 inhabitants in 2018, and 85% of tramadol prescriptions were combinations with paracetamol (see Supplementary Material S3). Fentanyl use rose from 4.5 to 15.9 prescriptions/100 inhabitants/year, while tapentadol, launched in 2011, reached a volume of 8.1 prescriptions/100 inhabitants in 2018 (Figures 2A,B).

**TABLE 1:** Evolution of opioid prescriptions in the region of Valencia, Spain, by year and active substance, period 2010–2018.

		Number (column %) of opioid prescriptions per year									
Opioid		2010	2011	2012	2013	2014	2015	2016	2017	2018	Total
Buprenorphine		115,265	119,723	122,744	129,593	135,986	136,659	127,991	114,957	104,128	1,107,046
		4.6%	4.0%	3.7%	3.5%	3.2%	3.1%	2.9%	2.4%	2.1%	3.1%
Codeine		286,408	329,403	341,630	343,588	339,722	397,168	422,045	482,848	539,212	3,482,024
		11.3%	10.9%	10.2%	9.2%	8.0%	9.1%	9.4%	10.2%	11.0%	9.9%
Fentanyl		190,275	244,436	297,891	436,700	561,439	608,603	612,621	633,319	646,475	4,231,759
		7.5%	8.1%	8.9%	11.7%	13.3%	13.9%	13.7%	13.4%	13.2%	12.0%
Hydromorphone		14,434	16,734	17,411	13,190	10,854	9,723	8,192	7,218	6,604	104,360
		0.6%	0.6%	0.5%	0.4%	0.3%	0.2%	0.2%	0.2%	0.1%	0.3%
Morphine		22,460	32,599	36,808	55,097	60,625	66,569	62,919	63,821	65,196	466,094
		0.9%	1.1%	1.1%	1.5%	1.4%	1.5%	1.4%	1.4%	1.3%	1.3%
Oxycodone		34,992	66,559	106,089	148,883	192,045	211,050	221,601	227,004	220,003	1,428,226
		1.4%	2.2%	3.2%	4.0%	4.5%	4.8%	4.9%	4.8%	4.5%	4.0%
Tapentadol		—	2,892	24,253	55,637	112,716	187,901	248,742	291,003	331,781	1,254,925
		—	0.1%	0.7%	1.5%	2.7%	4.3%	5.5%	6.2%	6.8%	3.6%
Tramadol		1,860,625	2,218,403	2,413,779	2,543,081	2,814,093	2,758,657	2,779,465	2,893,846	2,980,726	23,262,675
		73.7%	73.2%	71.8%	68.3%	66.6%	63.0%	62.0%	61.4%	60.9%	65.8%
Total		2,524,459	3,030,749	3,360,605	3,725,769	4,227,480	4,376,330	4,483,576	4,714,016	4,894,125	35,337,109

**FIGURE 3:**
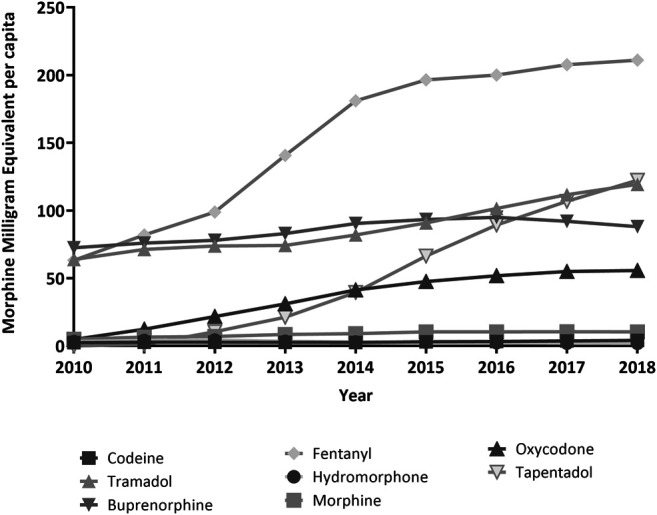
Trends in Morphine Milligram Equivalent per capita, period 2010–2018, region of Valencia, Spain.

Overall, the average dose prescribed per capita nearly tripled over the study period, from 215.4 MME/c in 2010 to 613.1 MME/c in 2018. For fentanyl, MME/c more than tripled, accounting for 34.4% of total MME/c in 2018. Morphine and tramadol dosing per capita increased 2-fold, and oxycodone, 10-fold. Tapentadol showed the highest growth rate of all the included drugs, overtaking tramadol as the second contributor in terms of MME/c by the end of the period (Figure 3; Table 2).

**TABLE 2:** Yearly dosage in terms of morphine milligram equivalent per capita (MME/c), total and per active substance, and % change from 2010 to 2018, region of Valencia, Spain.

Opioid	MME/c prescribed per year									% Change
	2010	2011	2012	2013	2014	2015	2016	2017	2018	
Buprenorphine	72.5	76.0	78.0	83.0	90.4	93.4	95.0	92.1	88.1	1.21
Codeine	2.5	2.8	2.8	2.7	2.6	3.1	3.3	3.7	4.1	1.64
Fentanyl	63.3	82.0	98.9	140.7	181.0	196.5	200.1	207.7	211.1	3.33
Hydromorphone	3.3	4.1	4.4	3.5	3.0	2.8	2.4	2.1	2.0	0.59
Morphine	5.0	6.6	7.1	8.5	9.1	10.4	10.4	10.5	10.4	2.08
Oxycodone	4.8	12.4	21.7	31.1	41.4	47.6	51.9	55.0	55.7	11.57
Tapentadol	0.0	1.1	10.5	21.2	39.5	66.4	89.3	106.8	122.4	107.54
Tramadol	63.9	71.3	73.8	74.3	82.0	90.8	101.5	111.7	119.4	1.87
Total	215.4	256.2	297.2	365.0	449.0	511.0	553.8	589.6	613.1	2.85

**FIGURE 4:**
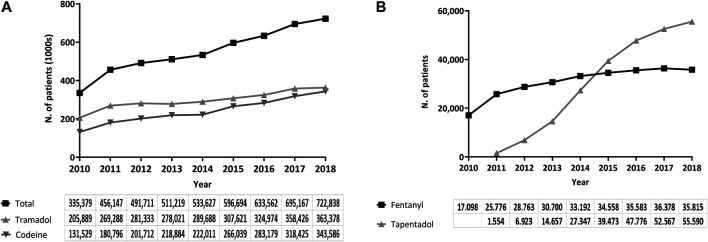
**(A,B)** Number of patients treated yearly, period 2010–2018, region of Valencia, Spain.

The annual number of patients receiving an opioid prescription more than doubled, and the number of patients treated every year with codeine grew by more than 2.5-fold. Similarly, 76% more patients were treated with tramadol in 2018 compared to 2010. The yearly number of patients treated with fentanyl also doubled. The number of patients treated with tapentadol reached 55,590 in 2018 since its launch in 2011. All in all, 2,107,756 patients received at least one opioid prescription over the study period (Figure 4A,B).

## Discussion

Our study, which included virtually all the adult population in the region of Valencia, shows that the annual volume of opioid prescriptions and the number of patients treated doubled over nine years, while dosage per capita tripled. Some specific trends underlie this general evolution and merit closer attention. First, there was a larger increase in dosage (MME/c) than in prescriptions per capita as a result of the relative increase in the prescription of strong vs. weak opioids. Together, fentanyl, oxycodone and tapentadol accounted for 6.8% of the total prescriptions in 2010, compared to 24.5% in 2018, whereas tramadol reduced its relative weight in total opioid consumption from 73.7% to 60.9%, despite its strong growth in absolute terms, from 1.86 to 2.98 million prescriptions. Second, there was a large increase in tramadol prescriptions, especially in combination with paracetamol. This pattern of treatment may be emerging as a substitute for non-steroidal anti-inflammatory drugs and strong opioids due to the growing concern about their risks. Whatever the reason, this finding suggests a generalized perception in our setting of tramadol as “opioid-lite,” with minimal side effects or propensity for addiction and misuse (Stannard, 2019); this perception contrasts with growing evidence of its adverse effects (Fournier et al., 2015; Zeng et al., 2019; Wei et al., 2020) and addictive potential (Thiels et al., 2019). Third, from 2016 the curve of annual opioid treatments started to flatten, mainly due to the slow-down in tramadol and fentanyl prescriptions. The decline, and least in fentanyl use, may be a response to the implementation of two relevant interventions targeting immediate-release formulations of this drug: a review of utilization in January 2016 (Instruction to review immediate-release treatments of fentanyl, 2016) and an AEMPS warning issued in February 2018 (Agencia Española de Medicamentos y Productos Sanitarios, 2019). However, appropriate methods should be employed to specifically analyze this association. Finally, the number of patients treated yearly doubled from 2010 to 2018. Even if the patients treated with strong opioids like fentanyl doubled, or grew sharply in the case of tapentadol, the main driver of the increase in patient numbers was the dramatic rise in the use of codeine as a fixed-dose combination with paracetamol and ascorbic acid. This combination has been used as a substitute for flu symptoms treatments since 2012, when the Spanish NHS stopped funding other formulations indicated for “minor symptoms” (Disposiciones generales Ministerio de SanidadServicios Sociales e Igualdad, 2012). Although this increase hardly contributes to the rise in MME/c due to the low codeine content of the formulation, the practice suggests a pattern of potentially unnecessary exposure to opioids.

To our knowledge, this is the first population-based study to describe real-world trends in volume, dosage and population exposure to prescription opioids in Spain. Reports from the AEMPS and some other local studies also provide information on consumption in the Spanish NHS, but with an important caveat: these studies usually report results as defined daily dose (DDD)/1,000 inhabitants/day. This is a problematic measure when applied to the field of opioids because a significant proportion of the opioid treatments are short-term treatments, and, unlike chronic medication, it is difficult to transform these figures into exposed people. Moreover, DDDs do not adequately characterize the intensity of the exposure to opioids of a population: for instance, whereas morphine DDD is set at 100 mg/day and tramadol is set at 300 mg/day, in terms of MME, 300 mg of tramadol equals only 30 mg of morphine (1 mg tramadol = 0.1 MME of morphine). Thus, our use of MME provides a more accurate view of trends and magnitudes.

Our findings are, in general, consistent with those obtained in different European countries, where a general pattern of growth in opioid consumption since the 1990s has been described. However, there are some notable variations in the relative levels of consumption between countries and some distinct, context-specific patterns of use for some drugs. For instance, with regard to the growth of the MME/c—one of the most striking findings in our study—the large differences in terms of exposure, trends and drivers of growth between regions suggests that utilization patterns may be strongly influenced by context-specific factors (Dzierżanowski and Ciałkowska-Rysz, 2017; Piper et al., 2018; Verhamme and Bohnen, 2019). Nevertheless, common usage patterns are clearly apparent throughout Europe. Studies in France, the United Kingdom, the Netherlands, Germany and some Nordic countries all show a steady increase in the use of opioids, the volume of population exposed to opioids and the amount of prescribed opioids per person. With regard to the main drugs contributing to the upward trend, fentanyl and tramadol are the greatest contributors, while oxycodone and tapentadol use (where launched) also show a sharp increase. Also, in the last ten years, most European countries show a stabilizing pattern in the volume of opioids prescribed (Chenaf et al., 2019; Hider-Mlynarz et al., 2018; Bosetti et al., 2019; Kalkman et al., 2019).

Our findings justify further research on the patterns underlying these trends, their appropriateness and their association with health and safety outcomes in our context. For instance, many clinical and non-clinical factors may be driving this growth, such as the evolution of population morbidity during the study period, changes in patterns of pain management and drivers of prescription, the impact of national and local warnings related to the use of opioids, and the influence of organizational components or promotional activities, among others. In light of the international evidence from North America and Europe, it is crucial to strictly assess the appropriateness of opioid prescriptions and manage them as a public health priority. Indeed, inappropriate prescriptions for opioids are associated with worse quality of life and a higher risk of negative outcomes like mental health disorders, overdose, overuse, death, and illicit opioid use (Bohnert et al., 2011; Kolodny et al., 2015; Rummans et al., 2018; Bohnert & Ilgen, 2019).

Our study has some limitations. First, the VID databases gather real-world clinical practice data and contain information as registered by health professionals during routine clinical practice, but data are not specifically prepared for research. In this sense, studies based on real-world clinical information like the VID are at risk of well-known biases such a differential recording, misclassification bias or missing data. However, prescription and dispensation information (the essential data in this study) is of the highest quality, as it is used for billing purposes. Second, we described prescription trends for all opioids by active substance, irrespective of formulation. However, an appreciable number of prescriptions are combinations of paracetamol plus low-dose tramadol or codeine, mainly used to treat common cold or flu-like symptoms. In fact, more than 20 million prescriptions in the study period correspond to paracetamol-tramadol combinations (see Supplementary Material S3). Even if this pattern may be concerning and warrant further examination, it poses different problems from a public health perspective than a rise in the use of major opioids, and these phenomena should be interpreted differently. Third, we did not distinguish between prescriptions for oncologic pain, chronic non-oncologic pain or acute non-oncologic pain, or between patterns of prescription of different specialists, and we did not examine clinical prescription patterns of interest, such as the use of high-strength formulations based on doubtful indications or prescription patterns that are not widely recommended by clinical guidelines, such as initiation of treatment with a long-term prescription or overlapping medications (Ministerio de Sanidad and Servicios Sociales e Igualdad, 2015). Instead, our study calls attention to potential areas of concern where research and intervention should be oriented in the short term. Finally, the generalization of our results to other settings outside Spain, or even to other Spanish regions, should be approached with great caution, as contextual factors may play an important role in prescription patterns.

Our results show that more than two million patients received 35 million prescriptions for opioids from 2010 to 2018. Both the yearly volume of opioid prescriptions and the number of patients doubled over this period, while dosage per capita in terms of MME/c tripled. Some specific prescription trends call for further attention. Even if proportions seem still far from epidemic, our findings warrant urgent research on the observed patterns of use, their appropriateness and their association with health and safety outcomes, especially for high-use and high-strength drugs.

## Data Availability Statement

“The datasets presented in this article are not readily available because legal restrictions on sharing the data set apply as regulated by the Valencia regional government by means of legal resolution by the Valencia Health Agency [2009/13312] which forbids the dissemination of data to third parties (accessible at: http://www.san.gva.es/documents/152919/157920/resolucionsolicituddatos.pdf). Upon request, authors can allow access to the databases in order to verify the accuracy of the analysis or the reproducibility of the study. Requests to access the datasets should be directed to Management Office of the Data Commission in the Valencia Health Agency (email: solicitud_datos@gva.es; telephone numbers: +34 961-928207; +34 961-928198)” Requests to access the datasets should be directed to “solicitud_datos@gva.es”.

## Ethics Statement

The studies involving human participants were reviewed and approved by Ethics Committee for Drug Research of the “Hospital Clínico-Universitario de Valencia” (Reference: F-CE-GeVA 14 v1.2; 2019, March 21). Written informed consent for participation was not required for this study in accordance with the national legislation and the institutional requirements.

## Author Contributions

IH, AG, SP, and GS were responsible for the study concept, design and data acquisition. IH carried out the data preparation and the statistical analysis, and AGS drafted the manuscript. IH, AG, SP, GS participated in the analysis and interpretation of data as well as the critical revision of the manuscript for important intellectual content. They approved the final version submitted for publication and agree to be accountable for all aspects of the work in ensuring that questions related to the accuracy or integrity of any part of the work are appropriately investigated and resolved.

## Funding

This study was funded by the 2018 Collaboration agreement between FISABIO, a research body affiliated with the Health Department of the Valencia Government, and Grünenthal Pharma S.A., to conduct independent research on “Patterns of use of opioids in the National Health System.” The funding sources had no access to the study data nor did they participate in the design or conduct of the study, data analysis, decisions regarding the dissemination of findings, writing of the manuscript, or decisions about its publication. The views presented here are those of the authors and not necessarily those of the FISABIO Foundation, the Valencia Ministry of Health, or the study sponsors.

## Conflict of Interest

The authors declare that the research was conducted in the absence of any additional commercial or financial relationships that could be construed as a potential conflict of interest.
