# Supplementary material for: Increasing Trends in Opioid Use From 2010 to 2018 in the Region of Valencia, Spain: A Real-World, Population-Based Study
Source: Front Pharmacol. 2020 Dec 11;11:612556. doi: 10.3389/fphar.2020.612556 (PMC7759684; doi:10.3389/fphar.2020.612556)
Supplement: Supplementary file 1 [file datasheet1.docx]

**Increasing Trends in Opioid Use from 2010 to 2018 in the Region of Valencia, Spain: a Real-World, Population-based Study.**

Hurtado Isabel, García-Sempere Aníbal, Peiró Salvador, Sanfélix-Gimeno Gabriel

**Supplementary Material**

**Table S1. Population of the region of Valencia, Spain, aged 18 years and over for the period 2010 to 2018.**

| **Year** | Population |
| --- | --- |
| **2010** | 4,204,320 |
| **2011** | 4,208,333 |
| **2012** | 4,219,929 |
| **2013** | 4,206,177 |
| **2014** | 4,107,580 |
| **2015** | 4,086,283 |
| **2016** | 4,065,930 |
| **2017** | 4,049,855 |
| **2018** | 4,073,170 |

**Table S2. Morphine Milligram Equivalent (MME) conversion table**

|  | |
| --- | --- |
| **Opioid (strength in mg except where noted)** | **MME Conversion Factor*** |
| Buprenorphine, transdermal patch (MCG/HR) | 12.6 |
| Buprenorphine, tablet or film | 30 |
| Buprenorphine, film (MCG) | 0.03 |
| Codeine | 0.15 |
| Fentanyl, buccal/SL tabet or lozenge/troche (MCG) | 0.13 |
| Fentanyl, film or oral spray (MCG) | 0.18 |
| Fentanyl, nasal spray (MCG) | 0.16 |
| Fentanyl, transdermal patch (MCG/HR) | 7.2 |
| Hydromorphone | 4 |
| Morphine | 1 |
| Oxycodone | 1.5 |
| Tapentadol | 0.4 |
| Tramadol | 0.1 |
| Source: CDC 2016.  *To be used in the formula: Strength per Unit  X (Number of Units/ Days Supply) X MME conversion factor = MME/Day. Please see Documentation for additional information on using the formula with transdermal patches.  MCG: microgram; HR: hour | |

**Table S3. Number of prescriptions, total and %, per type of medication, period 2010-2018, region of Valencia, Spain.**

| Medication | Number of prescriptions | % |
| --- | --- | --- |
| Acid acetyl salicilic & Codeine | 144,891 | 0.4% |
| Acid acetyl salicilic & Codeine & Caffeine | 54,958 | 0.2% |
| Buprenorphine | 1,107,046 | 3.1% |
| Dexketoprophene & Tramadol | 223,320 | 0.6% |
| Fentanyl | 4,231,759 | 12.0% |
| Hydromorphone | 104,360 | 0.3% |
| Morphine | 465,973 | 1.3% |
| Morphine Clorhidrate | 121 | 0.0% |
| Oxycodone | 336,479 | 1.0% |
| Oxycodone & Naloxone | 1,091,747 | 3.1% |
| Paracetamol & Codeine | 559,559 | 1.6% |
| Paracetamol & Codeine & Ascorbic acid | 2,722,616 | 7.7% |
| Paracetamol & Tramadol | 19,608,365 | 55.5% |
| Tapentadol | 1,254,925 | 3.6% |
| Tramadol | 3,430990 | 9.7% |
| Total | 35,337,109 | 100.0% |
